# Retraction Note: Hepatic stellate cell mediates transcription of TNFSF14 in hepatocellular carcinoma cells via H_2_S/CSE-JNK/JunB signaling pathway

**DOI:** 10.1038/s41419-022-05464-7

**Published:** 2022-11-30

**Authors:** Yanan Ma, Shanshan Wang, Yongle Wu, Bihan Liu, Lei Li, Wenjing Wang, Honglei Weng, Huiguo Ding

**Affiliations:** 1 Department of Gastroenterology and Hepatology, Beijing You’an Hospital affiliated with Capital Medical University, Beijing, 100069 China; 2 Beijing Institute of Hepatology, Beijing You’ An Hospital, Capital Medical University, Beijing, 100069 China; 3 Department of Medicine II, University Medical Center Mannheim, Medical Faculty Mannheim, Heidelberg University, Mannheim, 68167 Germany

**Keywords:** Apoptosis, Liver cancer

Retraction Note to: *Cell Death & Disease* 10.1038/s41419-022-04678-z, published online 15 March 2022

The Editors-in-Chief have retracted this article because there is overlap within some of the images or data. In particular:

1. Figure 1D (panel D3) shares features with Fig. 1D (panel D4), as well as Fig. 2D (panel NaHS)
2. Figure 2C (panel LX-2+PPG) shares features with Fig. 4C (panel Si + LX-2)
3. Figure 2C (panel PPG) shares features with Fig. 4C (panel Si)
4. Figure 5D (Cytoplasm panel p-JunB) overlaps with Fig. 5E (Cytoplasm panel p-JunB)

In addition, Fig. 1D (panels D3 and D4), as well as Fig. 2D (panel NaHS) appear to have an irregularity within the image.

As a result, the Editors-in-Chief no longer have confidence in the veracity of these data.

Huiguo Ding agrees with the retraction but not with the wording of the retraction notice None of the other authors has responded to correspondence from the publisher about this retraction.

